# The Risk of Developing Alzheimer’s Disease and Parkinson’s Disease in Patients with Inflammatory Bowel Disease: A Meta-Analysis

**DOI:** 10.3390/jcm11133704

**Published:** 2022-06-27

**Authors:** Marta Szandruk-Bender, Benita Wiatrak, Adam Szeląg

**Affiliations:** Department of Pharmacology, Wroclaw Medical University, Mikulicza-Radeckiego 2, 50-345 Wrocław, Poland; benita.wiatrak@umw.edu.pl (B.W.); adam.szelag@umw.edu.pl (A.S.)

**Keywords:** inflammatory bowel disease, Alzheimer’s disease, Parkinson’s disease, neurodegenerative disease, gut-brain axis, meta-analysis

## Abstract

Recently, a growing body of research has linked gut microbiota dysbiosis to central nervous system diseases, such as Alzheimer’s disease (AD) and Parkinson’s disease (PD), and has suggested that AD and PD pathology may take its origin from chronic inflammation in the gastrointestinal tract. Thus, this study aimed to elucidate whether inflammatory bowel disease (IBD) is associated with a higher risk of developing AD and PD as compared to the non-IBD population by conducting a meta-analysis. A thorough search of Pubmed and Embase databases was performed to identify all relevant articles. The quality of included studies was assessed using the Newcastle-Ottawa Scale. The odds ratios (ORs) with 95% confidence intervals (CIs) were analyzed using a fixed-effect model. To assess publication bias and heterogeneity among the studies, Egger’s test and L’Abbé plots were used, respectively. A total of eight eligible studies were included in this meta-analysis. No significant heterogeneity or significant publication bias was detected. The risk of developing AD in IBD patients was higher than in non-IBD patients (OR = 0.37; 95% CI = 0.14–1.00; *p* = 0.05), and there was a relationship between the occurrence of AD and Crohn’s disease or ulcerative colitis (OR = 0.11; 95% CI = 0.04–0.30; *p* < 0.0001, OR = 0.14; 95% CI = 0.04–0.49; *p* = 0.0024, respectively). The risk of developing both of the most common neurodegenerative diseases, AD and PD, was also significantly higher in patients diagnosed with Crohn’s disease or ulcerative colitis (OR = 0.21; 95% CI = 0.09–0.49; *p* = 0.0003, OR = 0.25; 95% CI = 0.13–0.51; *p* = 0.0001, respectively). This meta-analysis revealed a higher risk of AD and PD among CD and UC patients compared to the general population. It may suggest a key role for the gut microbiota in the pathogenesis of not only Crohn’s disease and ulcerative colitis but also AD and PD. The identification of this potential risk may provide earlier preventive measures to be implemented to reduce comorbidity and mortality rate.

## 1. Introduction

The gastrointestinal tract contains about 10^14^ microorganisms (commonly named gut microbiota), which encompass at least 1000 different microbial species [1]. In addition to several other beneficial properties that the gut microbiota provides to the host, it plays a crucial role in the maturation and functioning of the host’s immune system. Concomitantly, the host’s immune system influences the structure and function of the gut microbiota [2]. This balanced, reciprocal interplay between the host and its gut microbiota (eubiosis) is crucial to maintaining host homeostasis. Any disruption of eubiosis (dysbiosis) may cause pathological consequences to the host and lead to numerous disorders, ranging from intestinal diseases, such as inflammatory bowel disease (IBD), to more systemic diseases, such as allergy, diabetes, or metabolic syndrome [1,2,3].

It is well known that gut microbiota dysbiosis is associated with IBD, which encompasses two main entities, Crohn’s disease (CD) and ulcerative colitis (UC). However, until now, no single microbe or microbial milieu has been proven causal [4]. Intestinal microbiota differs qualitatively and quantitatively in IBD patients compared to that of healthy controls. It is characterized by an increase in the number of mucosa-associated bacteria, a decrease of short-chain fatty acid (SCFA)-producing bacteria, and a reduction in the overall microbial diversity [5,6]. Dysbiosis affects the host’s immune system and the integrity of the intestinal barrier. It may cause an inappropriate mucosal immune response and intestinal inflammation leading to the onset of IBD in genetically susceptible individuals. Mucosal epithelial barrier disorders, which result in the gut microbes’ translocation, promote the hyperactivation of the mucosal immune system and the production of proinflammatory cytokines, which contribute to stoking the inflammation observed in IBD patients [4,7].

Many recent studies revealed that the gut microbiota plays a significant role in regulating the bidirectional communication between the gut and the brain, commonly known as the gut-brain axis. The mechanisms enabling gut microbes to communicate with the brain and the brain with the gut are complex and largely undiscovered, including endocrine, immune, neural, and metabolic pathways [8]. Endocrine messages are carried by gut hormones, and immune messages by cytokines [9], whereas the vagus nerve is a crucial route of the neural connection of gut microbiota and the central nervous system (CNS). Molecules, such as SCFA, microRNA (miRNA), small non-coding RNA (sncRNA), and neurotransmitters are believed to be responsible for this communication [10]. All these signaling pathways may be affected by gut microbiota; however, it is postulated that gut microbiota influencing neurological and immune pathways contributes to the AD pathology, while gut microbiota influencing endocrine and immune pathways contributes to the PD pathology [9]. Thus, in addition to intestinal disorders, alterations of these bidirectional communications in the state of dysbiosis may contribute to the pathogenesis of CNS disorders, including the most common neurodegenerative disease, Alzheimer’s disease (AD) [11], and the second most common neurodegenerative disease, Parkinson’s disease (PD) [12]. The cardinal neuropathological feature of AD is the accumulation of the protein fragment β-amyloid (β-amyloid plaques) outside neurons and the accumulation of an abnormal form of tau protein (tau tangles) inside neurons [13], whereas intracellular aggregates of α-synuclein are pathological hallmarks of PD [14]. Previous studies have already found a link between abnormalities in the gut-brain axis and misfolding and aggregation of α-synuclein in PD [15,16], as well as between IBD and PD [17,18]. Recently, a growing body of research has shown that the aberrant gut microbiota-to-CNS pathway also causes β-amyloid deposition in AD [15,19] and that neuroinflammation is one of the main mechanisms linking microbiota to AD [19]. The gut microbiota plays a vital role in the activation of microglia, and it has been pointed out that manipulation of the gut microbiome, particularly with SCFA-producing bacteria, could modulate neuroimmune activation [15,20]. It is also not without significance that the permeability of two barriers increases with age—the intestinal wall barrier and the blood-brain barrier (BBB). This may also affect the communication of the intestinal microflora with the CNS and increase the exposure of the latter to potentially harmful particles produced by the intestinal microbiota [8,10]. Although the role of the gut microbiota in the pathogenesis of neurodegenerative disorders is only just beginning to emerge, there is a growing amount of experimental and clinical data suggesting that intestinal inflammation may contribute to its pathogenesis, as well as showing that neurodegenerative disorders, including AD and PD, may start in the gastrointestinal system even years before any other symptoms develop [21].

The current study aimed to meta-analyze the association of CD and UC with such neurodegenerative disorders as AD and PD, with an emphasis on AD as the most common neurodegenerative disease and the most common cause of dementia and comparing the occurrence of AD and PD between the IBD patients and non-IBD population. It is estimated that of the approximately 50 million people worldwide with dementia, 60–70% suffer from AD [13,22]. Knowledge of the coexistence of these diseases may indicate new approaches to the prevention and diagnosis of AD and PD and improve the effectiveness of their therapies.

## 2. Methods

### 2.1. Search Strategy

The meta-analysis was conducted following the guidelines of PRISMA (Preferred Reporting Items for Systematic Reviews and Meta-Analysis) [23] as well as in accordance with Cleophas’ guidelines [24]. Until the beginning of December 2021, two researchers (M.S.-B. and B.W.) independently analyzed the articles available in the PubMed and Embase bibliographic databases. The search aimed to collect a complete set of original research articles on IBD, including CD and UC, and neurodegenerative diseases focusing on AD and PD. The search terms included “Inflammatory Bowel Disease”, “Crohn’s Disease”, “Ulcerative Colitis”, “Neurodegenerative Disease”, “Alzheimer’s Disease”, and “Parkinson’s Disease”. The basis for the selection of articles for full-text analysis were their titles and abstracts. References in papers found in this way were also analyzed manually to identify other eligible articles.

### 2.2. Inclusion and Exclusion Criteria

This meta-analysis included original articles published in English that are cohort, case-control, or cross-sectional studies appraising the coexistence of IBD and AD or IBD and neurodegenerative diseases, including AD and PD. Comments, case-reports, reviews, meta-analyses, studies assessing other than AD or PD neurodegenerative diseases, studies not evaluating exposure or outcome, studies without a control group, articles without available full-text, and cell/animal and genetic studies were excluded. All duplicate publications have been identified and removed. Two researchers (M.S.-B. and B.W.) independently assessed the eligibility of each selected article and any discrepancies were resolved via discussion involving a third scientist (A.S.).

### 2.3. Quality Assessment

The quality of the included studies was assessed according to the “star system” of the Newcastle-Ottawa Quality Assessment Scale in three aspects: selection of study groups, comparability of groups, ascertainment of outcomes, and exposure to cohort and case-control studies, respectively (range from zero to nine stars) [25], as well as according to the modified version of this scale for cross-sectional studies (range from zero to eight stars) [26]. Studies getting ≥7 stars were considered as high-quality, 4–6 stars as medium-quality, and <4 stars as poor-quality studies [27]. The quality appraisal was carried out by two researchers (M.S.-B. and B.W.) and any discrepancies were resolved via discussion involving a third scientist (A.S.).

### 2.4. Data Extraction

A spreadsheet developed in Excel was used as a form for collecting data from articles. All researchers independently extracted the data. During the analysis of the articles, the following information was collected: paper title, name of the first author, publication year, country, years of the study, study type, participant number, and diagnosis method of IBD and neurodegenerative diseases.

### 2.5. Statistical Analysis

A meta-analysis of odds ratios was performed using TIBCO STATISTICA 13.3 PL software (StatSoft, Kraków, Poland). First, the confidence intervals (95% CI) were calculated, and on their basis, the *p*-values were obtained. Then, the significance level was set at *p* ≤ 0.05. To assess publication bias, Egger’s test was used. Due to the small number of included studies, funnel charts were not obtained. In small meta-analyses (approx. 7), the commonly used I2 heterogeneity statistic is considered imprecise and biased [28,29]. Therefore, given the small number of studies included, we decided to analyze the heterogeneity with L’Abbé plots.

## 3. Results

### 3.1. Search Results

**Figure 1:**
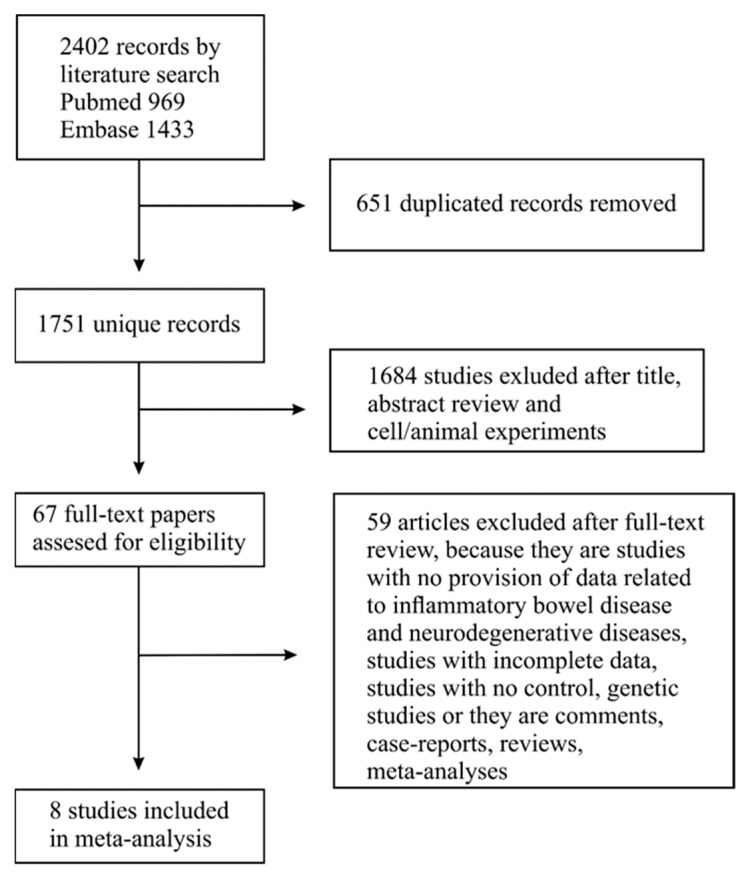
Flow diagram of the search process and study selection.

Searching for works (Figure 1) in two databases yielded 969 from PubMed and 1433 from the Embase database, respectively. After removing duplicates, there were 1751 papers. Of these, 1684 were excluded after screening the titles and abstracts due to non-compliance with inclusion criteria. The remaining 67 records were thoroughly evaluated. The review of the references in these papers did not allow any additional manuscripts to be included in the analysis. Another 59 reports out of 67 were excluded from the analysis because they did not contain data on the association between IBD and AD or IBD and neurodegenerative diseases, including AD and PD, or they did not provide data on a control group, or because of article type (they were comments, case-reports, reviews, meta-analyses). Finally, 8 articles were included in this meta-analysis [30,31,32,33,34,35,36,37].

### 3.2. Characteristics of the Included Studies

The main characteristics of the eight included studies are summarized in Table 1. Most of the selected studies were deemed of high quality, with a star score of ≥8. The selected papers were published in 2015–2020, reporting the results from Sweden, Denmark, Korea, Taiwan, USA, UK, and France. The total research period covered the years 1977–2018 and a population of close to 65 million participants. The included articles comprised five retrospective cohort studies, one prospective cohort study, one case-control study, and one cross-sectional study. Six studies (five cohort, one case-control) investigated the coexistence of IBD and neurodegenerative disease, including AD and PD, whereas three studies (two cohort, one case-control) evaluated AD incidences in the IBD population. Six studies (four cohort, one case-control, one cross-sectional) were designed to assess the relationship between both AD and PD, and CD, and four (two cohort, one case-control, one cross-sectional) to assess the relationship between AD and CD. Five studies (four cohort, one case-control) analyzed the relationship between both AD and PD and UC, and three (two cohort, one case-control) between AD and UC.

### 3.3. Association between IBD and Neurodegenerative Diseases

**Figure 2:**
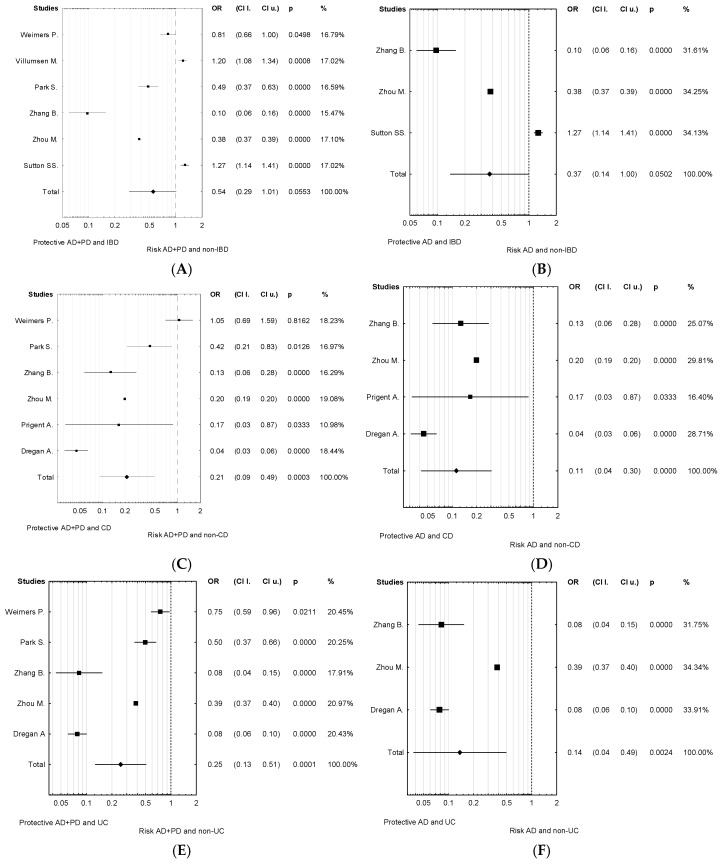
Meta-analysis result: forest plot diagrams depicting the association between neurodegenerative disease, including AD and PD and IBD (**A**); AD and IBD (**B**); neurodegenerative disease, including AD and PD and CD (**C**); AD and CD (**D**); neurodegenerative disease, including AD and PD and UC (**E**); AD and UC (**F**).

This meta-analysis showed that the risk of developing AD in the group of patients diagnosed with IBD was significantly higher than in non-IBD patients (OR = 0.37; 95% CI = 0.14–1.00; *p* = 0.050; Figure 2B), and the association was stronger in a subgroup of patients with CD or UC (OR = 0.11; 95% CI = 0.04–0.30; *p* < 0.0001, OR = 0.14; 95% CI = 0.04–0.49; *p* = 0.0024, respectively; Figure 2D,F). There was also a significantly higher risk of developing both of the most common neurodegenerative diseases, AD and PD, in patients diagnosed with CD or UC (OR = 0.21; 95% CI = 0.09–0.49; *p* = 0.0003, OR = 0.25; 95% CI = 0.13–0.51; *p* = 0.0001, respectively; Figure 2C,E). Subgroup analyses by age and sex revealed no statistically significant differences.

### 3.4. Evaluation of Publication Bias

The research of scientific articles was conducted without linguistic restrictions in two bibliographic databases: PubMed and Embase. The data for the analysis were collected independently by the authors of this study. This was to eliminate the risk of bias in the selection of articles. No significant publication bias was detected in Egger’s tests, the results of which are presented in Table 2.

### 3.5. Heterogeneity Analysis

**Figure 3:**
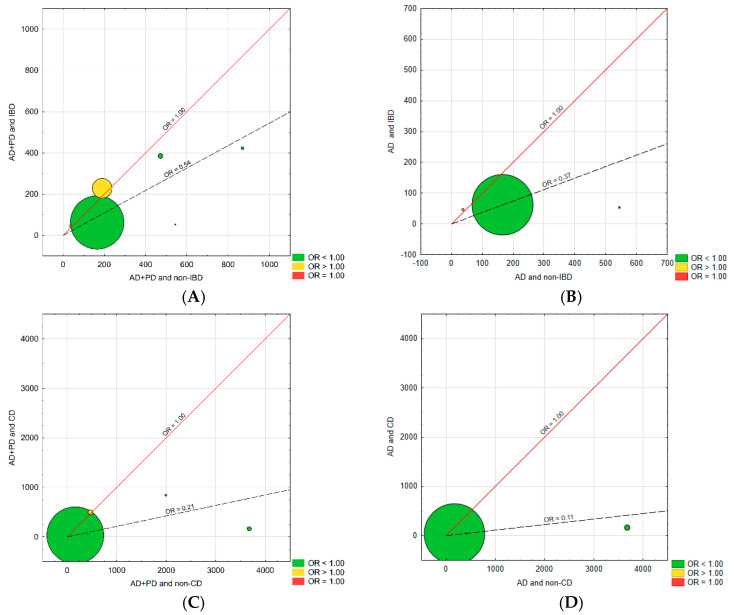

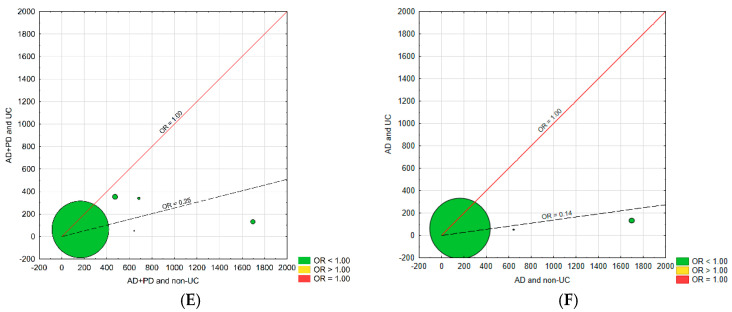
The L’Abbé chart—the relationship between the risk of neurodegenerative disease (AD + PD) in the group of patients with IBD and the control group (**A**); AD in the group of patients with IBD and the control group (**B**); neurodegenerative disease (AD + PD) in the group of patients with CD and the control group (**C**); AD in the group of patients with CD and the control group (**D**); neurodegenerative disease (AD + PD) in the group of patients with UC and the control group (**E**); AD in the group of patients with UC and the control group (**F**).

L’Abbé plots were used to assess the heterogeneity of the analysis (Figure 3). These plots show the dependence between the risk of AD and PD occurrence or only AD in the group of patients with IBD, CD, or UC and the risk of neurodegenerative disease in the group of patients without IBD. There was no significant variability in the results.

## 4. Discussion

Although the gut-brain axis has recently become the subject of many studies, there is little epidemiological research investigating the association between IBD and neurodegenerative diseases, especially AD. Therefore, this association is the subject of the current meta-analysis. This meta-analysis has demonstrated that IBD patients are at a greater risk of developing AD compared with the general population. Both CD and UC increase the risk of developing AD. It implies that CD or UC occurrence may be an important risk factor for AD development. Our results have also shown that the risk of developing of two most common neurodegenerative diseases, AD and PD, is higher in the group of patients diagnosed with CD or UC; however, the association is weaker than considering AD only. In the studies of Weimers et al. and Park et al. [32,33], in large Swedish and Korean cohorts, patients with IBD, in general as well as subgroups of patients with CD or UC, exhibited a significantly higher risk of PD compared to those without IBD. It is consistent with previous findings reported that there is an association between neurodegenerative diseases and IBD, especially for CD and UC [17,18,38].

Despite the small number of studies included in this meta-analysis, the number of patients was largely due to the population nature of the selected studies. The population extent covered by this study was close to 65 million, and the period covered was 1977 to 2018. Additionally, nearly all included studies were cohort studies. Cohort studies can be considered superior to case-control studies regarding calculating the estimated risk and the incidence of a specific disease [39]. Thus, the present study is appropriate to examine the incidence of AD and PD in the IBD population.

Significantly increased risk of neurodegenerative diseases, including ADand PD among CD and UC patients compared to the general population, revealed based on the quantitative synthesis of epidemiological studies, suggests the role of the gut-brain axis in neurodegenerative disorders. Characteristic features of IBD include chronic inflammatory state, gut microbiota dysbiosis, and impaired intestinal epithelial barrier, all of which may presumably contribute to neurodegenerative diseases development. As mentioned above, gut microbiota can influence immune signaling pathways, thus contributing to both AD and PD pathologies [9]. Hyperstimulation of the immune system, especially in an elderly population, leads to a chronic, low-grade state of inflammation, so-called inflammaging. It may be linked with the persistent inflammatory state of the gut mucosa elicited by age-related gut microbiota alterations involving its decreased diversity and stability. This may result in intestinal epithelial barrier impairment and an increase of proinflammatory cytokines and bacteria-derived products in the circulation with subsequent BBB impairment and neuroinflammation. Microbiota is able to influence the activation of peripheral immune cells and cytokine profile, which affect systemic and CNS inflammation and injury [8,9,16,40]. As demonstrated by Park et al. and Sutton et al., there was a lower risk of developing AD or PD in IBD patients who were under anti-inflammatory or immunomodulatory treatment [33,34].

Several plausible mechanisms may predispose to AD occurrence in IBD patients. Recently, neuroinflammatory processes are considered to play a key role in the pathogenesis of AD, with a growing emphasis on the role of the microglia, the brain’s resident immune cells [8]. Accumulation of β-amyloid triggers an immune response, which drives neuroinflammation and neurodegeneration in AD [40]. The growing body of evidence has suggested that systemic inflammation, e.g., in the course of CD or UC, can lead to neuroinflammatory changes and chronic microglial activation with the resultant release of a variety of proinflammatory products, including reactive oxygen species and cytokines, and the deposition of misfolded proteins in AD. Zhou et al. [37] in the case-control study showed that systemic inflammatory diseases, which are at least partially caused by the production of tumor necrosis factor-α (TNF-α) by activated macrophages, increase the risk of AD. TNF-α produced systemically may promote neuroinflammation in the brain via TNFR1 and TNFR2 receptor-mediated transcytosis [41]. Intriguingly, in the same study, TNF-α blocking agents decreased the risk for comorbidity AD in patients with various chronic inflammatory disorders but not IBD [37]. It may suggest that other cytokines besides TNF-α also play a role in neuroinflammation. Interleukin (IL)-1β, IL-6, and transcription factor NF-κB are molecules of great importance in the inflammatory process of IBD and have been involved in AD as well. Some studies have suggested that AD patients’ expression of proinflammatory cytokines is higher than in healthy controls [40].

Another possible mechanism by which CD and UC may prompt the occurrence of AD is gut microbiota dysbiosis. It has been demonstrated that microglial activation is under constant regulation by the gut microbiota and bacterial metabolites, especially SCFA. The lack of a complex host microbiota may lead to defects in the maturation, differentiation, and function of microglia [20]. Many studies have shown that both forms of IBD are associated with gut microbiota dysbiosis [1]. Both CD and UC are associated with reduced diversity of the intestinal microbiota, a decrease of SCFA-producing bacteria (especially *F. prausnitzii*), a decrease of bacteria with an anti-inflammatory capacity, and an increase of bacteria with a proinflammatory capacity [1]. The alterations of gut microbiota, like those in IBD, have also been shown to be correlated with the development of AD via neurological, endocrine, and immune pathways [8]. The shifted composition of the gut microbiota in both AD and PD patients includes lower proportions of vital SCFA-producing genera, such as *Butyrivibrio*, *Eubacterium*, *Clostridium*, and species *F. prausnitzii* [42]. The diminished presence of *F. prausnitzii* and *Eubacterium rectale* as well as increased abundance of *Escherichia*, and *Shigella* seems to be closely related to the cerebral accumulation of ß-amyloid in AD [42,43]. Moreover, AD patients are characterized by a higher abundance of proinflammatory bacteria, e.g., *Escherichia*, *Shigella*, *Pseudomonas*, and a lower abundance of anti-inflammatory bacteria, e.g., *Bacillus fragilis*, *Bacteroides fragilis*, and *Eubacterium hallii* in comparison to healthy controls. The profusion of proinflammatory and suppression of anti-inflammatory taxa were associated with proinflammatory cytokine release and amyloid deposition in the brain [42,43]. Impaired intestinal wall and blood-brain barriers and gut microbiota alterations are also linked to PD pathology. An increased presence of *Lactobacillaceae*, *Barnesiellaceae*, and *Enterococcaceae* and a decreased presence of *Clostridium coccoides*, *Bacteroides fragilis*, and *Prevotellaceae* have been observed in PD patients compared with healthy controls [44,45].

It is known that systemic inflammation and gut microbiota dysbiosis can also weaken the functions of the intestinal epithelial barrier and the BBB, making them more permeable and allowing entry of peripheral immune cells into the brain. Braniste et al. [46] showed that the integrity of the BBB depends on appropriate gut microbiota composition, and SCFA are key metabolites in mediating this effect. The disturbed intestinal epithelial and blood-brain barriers and microbiome dysbiosis associated both with CD and UC may facilitate the passage of gut microbiota metabolites into the CNS [33]. Enormous quantities of bacterially derived amyloids and lipopolysaccharides (LPSs) may leak from the gastrointestinal tract, accumulate at the systemic and brain level, and therefore contribute to the pathogenesis of AD. It has been proposed that LPSs and amyloids may directly pass through a compromised barrier and indirectly pass through these protective physiological barriers via LPS and amyloid-triggered cytokines or other small proinflammatory molecules that are normally transited [19,47]. In addition, bacterially-derived LPSs and amyloids can increase activation of the NF-κB signaling pathway, further exacerbate the gut’s leakiness, and increase the levels of cytokines, such as IL-17 and IL-22, which are involved in the inflammatory process of AD [47] and IBD as well [6]. It has been recently shown that exposure to bacterial amyloid proteins in the gut may lead to priming of the immune system and enhancing immune response to endogenous production of neuronal amyloid in the brain. Microbiota-derived amyloid proteins are able to alter the structure of proteins (proteopathy) and enhance inflammation in the nervous system, thereby initiating or augmenting brain disease (so-called mapranosis). Microbiota-derived amyloid proteins may act as prion proteins via molecular mimicry and elicit cross-seeding, in which one amyloidogenic protein (tau, Aβ, α-syn, curli, prion) provokes another to adopt a pathogenic β-sheet structure [48,49].

In the case of CD, another potential explanation of the co-occurrence with AD is that both CD and AD patients have shared increased tau protein expression. In CD, the pathological process is not restricted to the epithelial lining. However, it reaches all components of the gastrointestinal wall including the enteric nervous system (ENS), whose structure and neurochemical properties are altered during CD [50]. Prigent et al. [35] demonstrated that in CD but not UC, patients’ levels of tau expression are increased in both the submucosal and myenteric plexus, and this upregulation is likely mediated through the Nrf2/NDP52 pathway. Other recent studies have also revealed that enteric neurons also express tau protein like their CNS counterparts [51,52]. It supports the existence of a close relationship between CD and AD. Interestingly, these findings are analogical with those obtained for α-synuclein, which is overexpressed in the ENS in CD [53,54].

At the stage of reviewing this paper, another report from the observational study was published, providing further evidence that the incidence of such neurodegenerative diseases as AD and PD is higher in IBD patients than in the non-IBD population. This study also reveals that among IBD patients, female sex is a risk factor, while living in an urban area is protective for AD development [55]. However, due to the probability of repetition of any participant between the previously published study by Park et al. [33] assessing the Korean population in 2010–2013 and the newly published study by Kim et al. [55] assessing the Korean population in the period from 2009 to 2011 we decided not to include the article by Kim et al. in the statistical analysis to avoid the probability of double counting the data, and instead to present the results obtained by the authors descriptively [24].

Despite the importance and originality of our results, this study has several limitations. One of the limitations of this study was the small number of studies included in the meta-analysis. However, on the other hand, we believe that a large number of patients in the selected studies can partially compensate for the small number of studies included and provide a reliable and comprehensive assessment of the relationship between IBD and neurodegenerative disorders, emphasizing AD. Moreover, due to the lack of data, we were unable to analyze the complex states of IBD or their different stages. Furthermore, not all selected studies presented data concerning smoking status, caffeine intake, family history, drug exposure, and brain injury, which are important factors during the pathogenesis of neurodegenerative disorders. Therefore, it might limit our ability to evaluate the relationship between IBD and neurodegenerative disorders. Further studies evaluating the complexities of the intestinal environment concerning neurodegenerative diseases and focusing on patient demographic data are warranted and needed to thoroughly elucidate the connection between the occurrence of IBD and neurodegenerative disorders.

## 5. Conclusions

In conclusion, the current meta-analysis showed a significant relationship between the occurrence of CD or UC and AD and PD in the adult population. It may imply the critical role of the gut microbiota in the pathogenesis of not only CD and UC but also AD and PD. Therefore, vigilance and education for these neurodegenerative diseases among patients with IBD may improve early recognition and intervention to slow cognitive decline and improve patients’ quality of life.

## Figures and Tables

**Table 1:** Main characteristics of eight studies included in this meta-analysis.

Study	Study Design	Country, Year	Study Period	AD, PD Cases among Study Participants	Newcastle-Ottawa Scale
Dregan A. et al. [30]	cohort study	UK, 2015	2002–2013	47 AD/7705 CD 93 AD/12,335 UC 266 AD/308,843 non-IBD	Selection: 4 Comparability: 2 Outcome/Exposure: 2
Villumsen M. et al. [31]	cohort study	Denmark, 2018	1977–2014	335 PD/76,477 IBD 39,784 PD/7,548,259 non-IBD	Selection: 4 Comparability: 1 Outcome/Exposure: 3
Weimers P. et al. [32]	cohort study	Sweden, 2018	2002–2014	23 PD/11,418 CD 69 PD/24,442 UC 103 PD/39,652 IBD 839 PD/396,520 non-IBD	Selection: 4 Comparability: 2 Outcome/Exposure: 3
Park S. et al. [33]	cohort study	Korea, 2019	2010–2013	92 PD/38,861 IBD 15 PD/12,631 CD 77 PD/26,230 UC 134 PD/116,583 non-IBD 19 PD/37,893 non-CD 115 PD/78,690 non-UC	Selection: 4 Comparability: 2 Outcome/Exposure: 3
Sutton S. et al. [34]	cohort study	USA, 2019	2010–2018	523 AD/24,057 IBD 1166 AD/42,255 non-IBD	Selection: 4 Comparability: 2 Outcome/Exposure: 2
Prigent A. et al. [35]	cross-sectional study	France, 2020	na	12 AD/16 CD 2 AD/16 non-CD	Selection: 2 Comparability: 1 Outcome/Exposure: 2
Zhang B. et al. [36]	cohort study	Taiwan, 2020	1995–2010	33 AD/1742 IBD 11 AD/584 CD 22 AD/1158 UC 32 AD/17,420 non-IBD 14 AD/5840 non-CD 18 AD/11,580 non-UC	Selection: 4 Comparability: 2 Outcome/Exposure: 3
Zhou M. et al. [37]	case-control study	USA, 2020	na	4440 AD/279,040 IBD 6160 AD/201,870 CD 2650 AD/168,870 UC 338,400 AD/55,954,070 non-IBD	Selection: 4 Comparability: 1 Outcome/Exposure: 3

**Table 2:** Publication bias calculated with Egger’s test.

Comparison	Egger’s Test (C.I._l._–C.I._u._)	*p*-Value
AD + PD vs. IBD	−16.88–27.89	0.49
AD + PD vs. CD	−5.57–5.81	0.96
AD + PD vs. UC	−17.66–11.64	0.56
AD vs. IBD	−205.36–218.23	0.77
AD vs. CD	−8.88–4.18	0.34
AD vs. UC	−70.47–51.63	0.30

Abbreviations: C.I._l_., lower confidence interval; C.I._u_., upper confidence interval.

## Data Availability

The data underlying this article will be shared on request to the corresponding author.
